# Circular citizenship behaviors: How individuals can promote systemic change toward a circular economy

**DOI:** 10.1016/j.isci.2025.112906

**Published:** 2025-06-14

**Authors:** Isabel Maria Pacheco, Ellen van der Werff, Linda Steg

**Affiliations:** 1 Faculty of Behavioural and Social Sciences, University of Groningen, Grote Kruisstraat 2/1, Groningen 9712 TS, the Netherlands

**Keywords:** Environmental science, Social sciences, Sociology

## Abstract

Sustainability challenges, such as transitioning to a circular economy, require people to engage in sustainable behaviors. Yet, current systems often inhibit sustainable behaviors, implying systemic changes are needed. Importantly, citizens can take action to provoke systemic changes. We propose the systemic change through citizen action (SCCA) framework that indicates which actions citizens can take to promote systemic changes that contribute to a circular economy. Specifically, the SCCA proposes that citizens can promote systemic change to support circularity by engaging in circular citizenship behaviors that aim at influencing other actors, including governments, businesses, and other citizens. The SCCA indicates which behaviors citizens can take to urge different actors to take action that would promote systemic change, thereby integrating previously dispersed research. We provide a research agenda indicating how the SCCA framework can inspire future research, including identifying key factors influencing citizens’ likelihood to engage in circular citizenship behaviors.

## Graphical abstract

**Figure:**
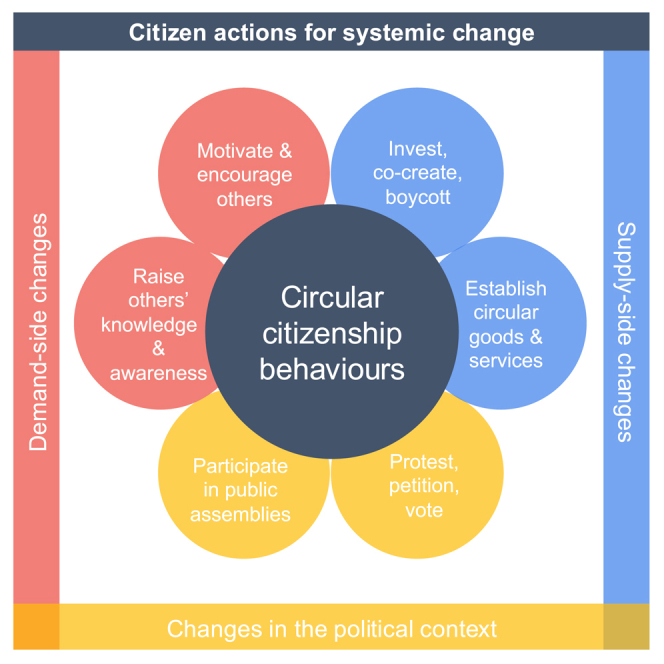

## Introduction

The age of the Anthropocene is characterized by increasing human impact on nature, causing rising temperatures, biodiversity loss, and natural disasters, such as floods, droughts, and wildfires.^1^ Water, air, and soil are polluted by industrialization, pesticides, urbanization, and poor waste management, which harms people’s health and increases poverty and inequality.^2^ Over 100 billion tons of raw materials enter the global economy annually, depleting natural resources and further exacerbating environmental problems.^2^ Vulnerable groups are being affected disproportionately, while they have contributed the least to such problems.^3^

One promising route to mitigate global challenges like climate change, biodiversity loss, depletion of resources, waste problems, and pollution is to move to a circular economy (CE).^4^ The CE shifts away from the traditional linear economic model of extracting new resources, using them to produce consumer goods, and eventually discarding them as waste.^5^ In contrast, the CE aims to decouple resource use from value creation through three pathways: *narrowing the loop* by reducing overall resource use, *slowing the loop* by keeping products in use for longer periods, and *closing the loop* by recycling or repurposing resources rather than discarding them as waste.^6^ Currently, we are still far away from a CE, with less than 10% of the world economy being circular.^7^ Global efforts to address climate change will be more effective when different actors take action simultaneously.^3^ Governments, civil society, and the private sector together can promote a shift to the CE.^3^ Transitioning to a CE requires action and collaboration across actors^8^^,^^9^^,^^10^^,^^11^^,^^12^ (for governments, see de Waal^13^; for businesses Geissdorfer and colleagues,^14^ Niessen and Bocken,^15^ and Urbinati and colleagues^16^; for citizens Camacho-Otero and colleagues^17^).

Firstly, businesses can shift their traditional linear approach toward a circular model, for example, by adopting circular business models and processes, such as innovative product-service systems (e.g., reuse platforms, pay-per-use models), longer warranties for electronics or electrical goods, or enhancing manufacturing through refurbishment, remanufacturing, or recycling.^18^^,^^19^

Secondly, governments can create supportive circumstances, such as implementing policies and incentives that facilitate and promote circular products and practices and/or inhibit and discourage linear products and practices. For example, China adopted the Circular Economy Promotion Law^20^ and released a five-year plan to develop the country’s CE through various initiatives.^21^ These include municipal waste recycling systems and policies to promote circularity in the remanufacturing industry.^22^ Similarly, in 2015, the European Commission adopted its first Circular Economy Action Plan, which was updated in March 2020.^23^ The action plan introduced policies promoting circular product design and processes, as well as policies aimed at encouraging circular consumption to prevent waste and keep resources in the economy as long as possible.

Thirdly, individuals can contribute to a CE by engaging in behaviors that contribute to and support the CE, such as engaging in circular consumption.^17^ Public discussions and scientific literature on the CE often focus on the role of producers, e.g., developing circular business models^6^^,^^11^^,^^24^^,^^25^ or circular supply chains.^26^^,^^27^ Fewer studies have focused on the role of individuals in the transition toward the CE. Research about the individual’s role mostly focuses on the role as a consumer and their circular consumption behaviors, such as reducing car use,^28^^,^^29^^,^^30^ downsizing to a smaller home,^31^ or co-housing.^17^^,^^32^^,^^33^ Research has identified a wide range of circular consumption behaviors, categorized in so-called R strategies^34^ (see Table 1). The R strategies follow a hierarchy from most impactful in achieving circularity and reducing environmental problems *(refuse)* to least impactful *(recover*).^34^

**Table 1:** Circular consumption in the 9R framework

Strategy	Description	Pathway
R0 Refuse	Make product redundant by abandoning its function or by offering the same function with a radically different product	Narrowing the loop
R1 Rethink	Make product use more intensive (e.g., by sharing product)	
R2 Reduce	Increase efficiency in product manufacture or use by consuming fewer natural resources and materials	
R3 Reuse	Reuse by another consumer of discarded product which is still in good condition and fulfills its original function	Slowing the loop
R4 Repair	Repair and maintenance of defective product so it can be used with its original function	
R5 Refurbish	Restore an old product and bring it up to date	
R6 Remanufacture	Use parts of discarded product in a new product with the same function	
R7 Repurpose	Use discarded product or its parts in a new product with a different function	
R8 Recycle	Process materials to obtain the same (high grade) or lower (low grade) quality	Closing the loop
R9 Recover	Incineration of material with energy recovery	

## Individuals as circular citizens

Individuals often struggle to adopt circular consumption behaviors depicted in Table 1, as current systems do not always support them. Even motivated consumers can face contextual barriers, such as low availability of a service (e.g., no repair shops nearby) or inadequate infrastructure (e.g., missing public transport connections). Hence, individuals’ engagement depends not only on their motivations and abilities but also on the context in which choices are made. Current systems often fail to provide individuals with the opportunities and incentives needed for circular consumption.^35^ Yet, the need for addressing such systemic barriers is often overlooked.^35^^,^^36^ Importantly, these systems are shaped by the actions of different actors—such as governments, industries, companies, and other consumers—whose choices determine the attractiveness and feasibility of circular consumption behaviors. Specifically, these actors can (and sometimes need to) take steps to make circular consumption behaviors more feasible and appealing—such as increasing access to circular goods or services (e.g., regenerative energy), implementing supportive policies, laws, or subsidies, or improving infrastructures (e.g., public transport and cycling paths), which can contribute to systemic changes. Does this mean that individuals need to passively wait for other actors to elicit systemic changes, or can they contribute to systemic changes themselves?

We propose that individuals can play a key role in promoting systemic changes by taking action to urge other actors to take steps that would increase the feasibility and attractiveness of different circular behaviors. That is, they can act upon their role as circular citizens (cf. Hobson^37^^,^^38^) rather than only taking up the role of circular consumers, which may be critical to bringing about the systemic changes needed to achieve a CE.^8^^,^^37^^,^^38^^,^^39^ We define *circular citizenship behaviors* (CCBs) as individual and collective actions that aim at influencing other actors to support circularity, thereby actively shaping the societal system in favor of narrowing, slowing, and closing loops of resource use. Through CCBs, citizens can increase the likelihood of systemic changes that make their own and others’ circular consumption behaviors possible and more attractive. Importantly, this implies that individuals are not merely passive consumers. They can actively help shape societal and economic systems. Through CCBs, citizens can drive systemic changes that majorly impact environmental quality. Systemic changes can encourage circular consumption behaviors of many people, likely making CCBs high-impact behaviors (cf. Hampton and Whitmarsh,^35^ Whitmarsh and colleagues,^36^ and Wei and colleagues^40^). Our proposition aligns with calls to place citizens at the core of a CE and to emphasize its social dimension, including calls for a circular society (e.g., Calisto-Friant and colleagues,^41^ Jaeger-Erben and colleagues,^42^ and Leipold and colleagues^43^) or a social circular economy.^8^^,^^44^ Similarly, it has been argued that individuals can contribute to sustainable transitions in multiple roles, next to being a consumer, including the role of investor, role model or influencer, organizational participant, or citizen.^3^^,^^35^^,^^37^^,^^45^^,^^46^^,^^47^

However, surprisingly, a systematic overview of the behaviors citizens can show to promote systemic change is missing. To address this gap, we propose the systemic change through citizen action (SCCA) framework, which we apply to the CE. This integrative framework is based on theorizing regarding which CCBs individuals can adopt to urge other actors, namely governments, businesses, and other citizens, to contribute to systemic changes supporting a CE. The SCCA framework builds on and integrates literature on individual actions to bring about social change, such as environmental citizenship behaviors,^48^^,^^49^^,^^50^ as well as collective action and environmental activism.^45^^,^^51^^,^^52^^,^^53^^,^^54^ Further, we build on literature on collaborative actions of citizens and other actors, including collaborative governance,^55^^,^^56^^,^^57^ co-design between businesses and citizens,^58^^,^^59^ and stakeholder engagement.^60^^,^^61^^,^^62^

**Figure 1:**
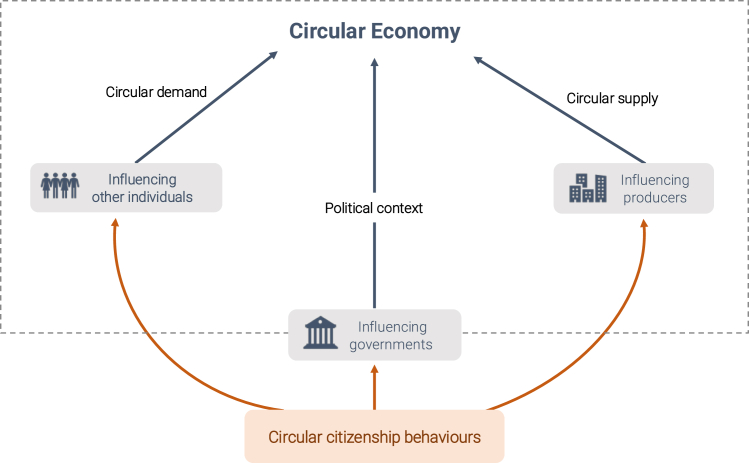
The systemic change through citizen action framework of circular citizenship behaviors and the three pathways through which individuals can promote systemic change toward a CE

The SCCA framework distinguishes three main pathways through which citizens can promote systemic change toward a CE. Each pathway targets a specific societal actor that can contribute to systemic change: other citizens, businesses, and governments, respectively. Firstly, citizens can encourage other citizens to adopt circular practices, shifting the demand toward circularity. Secondly, citizens can urge businesses to offer more circular goods and services or create circular solutions themselves, such as selling goods for reuse or establishing a community for sharing goods, shifting the supply toward circularity. Thirdly, citizens can advocate for laws, taxes, subsidies, or infrastructures that support CE practices, promoting the implementation of policies that support circularity. These three different pathways illustrate how citizens can transcend individual change and dismantle systemic barriers, making circular consumption more attractive and feasible for all (see Figure 1).

### Influencing other individuals to promote demand toward circularity

Citizens can influence other people to engage in circular consumption and citizenship behaviors, including people they know personally, such as friends, relatives, or colleagues, as well as the broader public. When successful, influencing other people to consume more circularly could shift the demand away from linear to circular goods and services, making them more attractive due to economies of scale. After all, the more other people engage in circular consumption behaviors, the more attractive and feasible it becomes for both businesses and consumers to engage in the CE.

Different types of social influence, such as social norms and role modeling, can stimulate others to engage in sustainable behavior (see, for example, a meta-analysis on social influence to encourage resource conservation^63^ and on factors related to recycling^64^). First, citizens can try to influence the thoughts and behaviors of people they know, for example, in face-to-face conversations and through posting messages or links on social media. Doing so could raise others’ knowledge and awareness about environmental problems that arise from the current linear economy and consumption, may lead to more favorable attitudes toward circularity and might motivate them to engage in circular behaviors.^47^^,^^65^ Next, individuals can point out ways to engage in consumption or citizenship behaviors and strengthen others’ beliefs that they can engage in these behaviors, which may increase feelings of self-efficacy.^66^ For example, explaining to a friend how to repair a good or telling them about a repair shop nearby that carries out these repairs could strengthen their self-efficacy, which makes it more likely they would repair instead of discard a good. Further, individuals might share their circular behaviors online via social media, write up tips and tricks for being more circular in blog posts, or upload tutorials on how to repair certain products or ways to reuse items. Further, affirming that circular behaviors contribute to reducing environmental problems could encourage others to engage in circular behaviors by improving their sense of outcome efficacy.^66^ Moreover, by expressing one’s approval of engaging in circular behaviors, such as using their bike or public transport, and disapproval of more linear behaviors, individuals might strengthen others’ injunctive norms to engage in circular behaviors. As role models, people can demonstrate circular behaviors by engaging in them themselves, which might help others learn how to engage in circular behaviors and strengthen descriptive norms (i.e., perceptions of how many others engage in circular behavior), in turn encouraging others to engage in the circular behaviors themselves as well.^63^^,^^67^ Exerting social influence on friends, family, or colleagues might therefore not only encourage circular behavior but also the underlying determinants, which make it more likely that changes last.

Second, citizens can participate in or support environmental organizations to influence the broader public. Environmental organizations can educate the broader public about the CE, demonstrate ways to engage in circular behaviors (e.g., Deelmobiliteit.nu shows which car-sharing options are available in one’s neighborhood^68^ and Milieucentraal lists a range of circular behaviors people can adopt^69^), or provide platforms where individuals and communities can share ideas of how to engage in circular behaviors.

### Influencing businesses or creating circular goods and services to improve the supply of circular goods and services

Citizens can influence businesses to improve their supply of circular goods and services, making circular consumption behaviors more attractive and feasible. First, citizens can use their role in the company they work for (as workers and professionals^45^) to call for more circularity in their organization. Particularly, professionals with decision-making power or professionals responsible for sustainability in their organization are likely to be impactful in this respect.^46^ For example, a person working at a phone manufacturer could push for using recycled instead of virgin materials in smartphones or develop a circular product-service system for smartphones. Importantly, employees in any sector or position can aim to promote circularity as part of their job. For example, HR employees could recruit people who want to integrate circularity into their positions. Similarly, employees might start a green initiative at work^70^ or promote circular work strategies and business processes, such as urging their organization to save energy, avoid plastic in the canteen, or reduce paper use,^71^^,^^72^^,^^73^ which fall under the CE’s R strategy of *reduce*.

Second, citizens as co-creators or *prosumers*^74^^,^^75^^,^^76^ can participate in the design processes of circular goods and services of other companies to voice consumers’ needs, demands, and wishes regarding circular products and services.^59^^,^^61^^,^^62^ Co-creation by businesses and customers can lead to more attractive and feasible circular goods and services by enhancing product quality^77^ and improving usage experience and brand loyalty.^78^^,^^79^ For example, citizens as co-creators can raise their concerns and needs regarding prices, warranties, or repair times during the design process of a smartphone product-service system (*rethink* strategy), which could help create an offer that is more attractive to consumers and, therefore, increases the likelihood of success of that circular product or service. Thus, co-creating can attract new customers and create value for circular businesses.^80^ Co-creation can also help generate original input for innovations,^81^^,^^82^ further advancing circular goods and services and the likelihood that they get taken up by consumers. Indeed, co-creation in circular food innovation can foster product and service innovation by making use of consumers’ ideas.^83^ Similarly, a recent literature review highlights customers’ potential role in advancing circular practices in fashion.^84^ To inform businesses about consumers’ preferences and needs, co-creators can share their ideas, for example, on organizations’ internet bulletin boards, by taking part in surveys, or attending stakeholder meetings.^85^ Hence, citizens as co-creators can engage in a range of different behaviors to urge businesses to increase and improve the supply of circular goods and services, which could make circular consumption more feasible and attractive.

Third, people can pressure businesses not aiming for circularity by boycotting these businesses by not buying their products and services. Boycotting implies refusing to purchase products or services from specific businesses or organizations to urge them to change, while not necessarily abstaining from a product or service generally (as in the R strategy *refuse*). For example, a consumer might boycott businesses that manufacture only virgin materials or that makes products that cannot be easily repaired, and rather buy the product from a company that supports circularity. Thus, boycotting is a form of activism^86^^,^^87^ and therefore CCB.

Fourth, citizens can influence companies toward more circularity through their investment choices if they have sufficient financial resources. As investors, citizens can buy shares of a company to gain influence in that company and urge it to become more circular by changing the business model or way of production. Furthermore, they can invest in pensions, (exchange-traded) funds, stocks, and bonds that support circularity, or have an account with a bank that invests their money in circular companies or projects. Interestingly, citizens are increasingly using their investor role as reflected in the rise of climate-related investment funds.^88^ Using one’s voice for investments can be impactful. For example, in 2024, one of the Netherlands’ largest pension funds, ABP, announced their new green investment policy for the coming years,^89^ indicating that from the invested pension assets of over 500 billion euros, at least 30 billion euros would need to go to impact investments to promote the energy transition, biodiversity, and innovation, among others. The decision was majorly influenced by pressure from ABP’s constituents, and following a survey among the pension fund’s members that indicated, among others, that members would even accept a lower pension to secure sustainable and responsible investments.

Fifth, citizens can establish circular structures themselves that directly improve the provision of circular goods or services and enable other citizens to engage in circular consumption behaviors (see also literature on citizen engagement in shaping systems of provision^36^^,^^38^^,^^90^). Citizens can either supply certain goods and services themselves or join or support organizations that do so. For example, citizens can organize clothes swaps, enabling the *reuse* strategy of circular consumption. Citizens can also set up a tool library in their street where everyone can bring their tools and borrow the ones that are brought in there by others. This enables the *rethink* strategy of circular consumption by sharing tools that are not often needed, instead of buying them. This can be done informally with neighbors and friends or more formally through platforms for lending and borrowing goods.^91^ Similarly, citizens can establish a community to share goods, such as a lawn mower or their car. Citizens can also participate in or support circular initiatives, such as repair cafés, where they can find tools and materials needed for repairing goods and are assisted in the repairs by volunteers, enabling the *repair* strategy of circular consumption.^92^ Such bottom-up initiatives can be effective.^70^ For example, community energy initiatives appear to promote the implementation of the R strategies of *reduce* and *rethink* in energy consumption^93^ and community repair contributes to enabling citizens, particularly marginalized groups, to be more circular.^94^ This shows that citizens who establish or support circular communities can influence others to become more circular. Such grassroots initiatives are argued to lead to a better balance between the currently disproportionately high influence of powerful businesses and institutions on one hand and self-reliant communities on the other.^8^ Some roles, such as upcycler, maker, and giver,^11^ may also be seen as creating circular goods for oneself or others, therefore influencing the supply of circular goods.

Sixth, citizens can support environmental organizations through volunteering or donating money that helps the organizations to urge businesses to become more circular, e.g., enabling them to lobby, educating businesses in designing more circular processes and products, and helping to establish multi-stakeholder dialogues for more circularity. Supporting non-governmental organizations that advocate for a CE can be very successful, as shown in the lawsuit of the environmental NGOs Milieudefensie/Friends of the Earth Netherlands. Encompassing more than 17000 citizens in total, they urged Shell to act in line with the Paris Climate Agreement and reduce its CO_2_ emissions by 45% by 2030 compared to 2010 levels and to net zero by 2050.^95^ The costs of this lawsuit, such as for lawyers and researchers, were covered through donations from the public.

### Influencing governments to implement policies that facilitate circularity

Citizens can urge governments to implement policies that can make it more feasible, attractive, and normative for businesses and people to engage in the CE. Governments can support the CE in various ways, such as changing infrastructure in favor of circularity, implementing laws, taxes, and subsidies that support the CE, investing in the development of sustainable technology, and educational campaigns. Through influencing governments, citizens can progress the political context to be supportive of a CE, which makes circular supply and demand more attractive and feasible and further contributes to systemic change.

To urge governments to implement such policies, first, citizens can protest, join public demonstrations, and participate in sit-ins, strikes or rallies to voice their needs and demands. Citizens’ engagement in mass movements can indeed be a catalyst for tackling climate change by influencing governments’ ambitions to tackle climate change.^3^

Second, citizens can sign petitions that promote circularity, write letters or emails or call government officials to urge them to promote circularity.

Third, they can participate in the political system to support circularity, e.g., by participating in public assemblies or hearings or being involved in the design of new policies that promote systemic change toward a CE.^58^^,^^96^ Public participation can inform decision-making processes with cultural values and knowledge, which enables accelerated actions toward sustainability.^3^ Fittingly, the European Commission has recommended in their 2020 Circular Economy Action Plan to include the CE as a regular theme in citizen dialogues.^23^

Fourth, citizens can use their votes in elections, e.g., on a local, national, or international level, to increase the likelihood that political parties that will advance a CE get into power. In 2022, Finland, Sweden, Denmark, and Austria scored highest on the Sustainable Development Goals within the EU.^97^ Of those countries, Finland, Sweden, and Austria all had green parties in their governing coalitions,^98^ suggesting that voting for green parties might indeed promote a CE.

Fifth, as for influencing other citizens and businesses, citizens can urge governments to advance circularity by participating in or donating to environmental organizations that lobby for circular policies or support infrastructures. An example is the activist group Extinction Rebellion in the Netherlands, which has protested against fossil fuel subsidies since 2021, culminating in an almost month-long road block of a major highway close to the Ministry of Economic Affairs and Climate Policy. These actions eventually led the Dutch Parliament to ask the cabinet to draw up a plan to phase out fossil fuel subsidies.^99^^,^^100^ Further, during the 28th UN Climate Conference in Dubai,^101^ the Netherlands set up an international coalition to phase out fossil fuel subsidies, led by the Dutch minister Jetten.^101^^,^^102^ These examples show how citizens can actively voice their demands for more circularity and influence other actors to support the CE transition.

**Figure 2:**
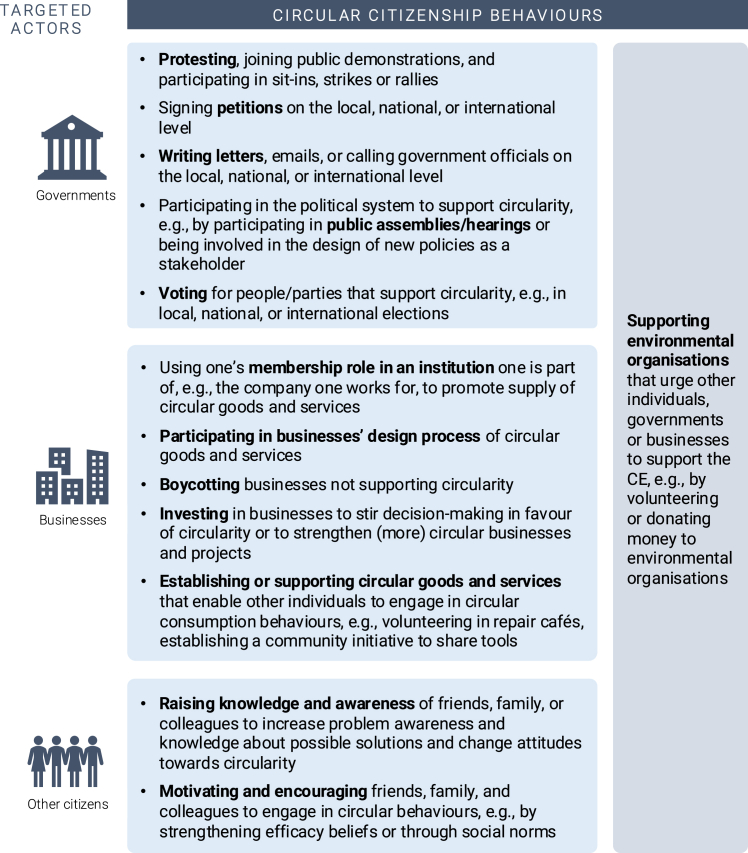
Overview of circular citizenship behaviors that contribute to systemic change

Figure 2 offers an overview of the various CCBs and the actors that they target to promote systemic change. CCBs allow citizens to actively influence the economic and societal system, accelerating the transition to a CE. They demonstrate that individuals need not wait for others to act; they can drive systemic change themselves, enhancing the feasibility, attractiveness, and normativity of circular consumption. However, currently, most people seem to hardly engage in CCBs.^35^^,^^103^

**Figure 3:**
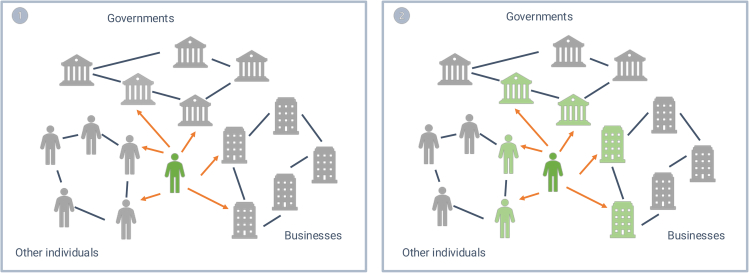
The influence of citizens on other actors through circular citizenship behaviors, creating broader systemic change Circular citizens influencing other actors are shown in green, their CCBs are represented by the orange lines. Targeted actors are shown in gray. When they are influenced by CCBs—either directly or indirectly—and become more circular, they turn light green.

Figure 3 shows how CCBs can influence other actors and create systemic change. By motivating other actors to support the CE, individuals may trigger ripple effects: the actions of the actors they influenced may inspire further change among others. For instance, individuals who adopt circular behaviors because they were urged to do so might help establish new social norms, motivating others to engage in similar actions (for example, see literature on social identity,^104^^,^^105^ dynamic norms,^106^^,^^107^^,^^108^ and social networks and social tipping points^109^^,^^110^^,^^111^). Governments that support circularity might led by example and encourage citizens and businesses to adopt circular practices.^112^ Businesses transitioning to circularity might demonstrate the feasibility and potential benefits of circularity to other businesses, inspiring them to follow suit. Government policies that support circularity likely facilitate businesses in becoming more circular, and more circular businesses are likely to make it more attractive and feasible for individuals to behave more circularly. In sum, over time, these ripple effects likely amplify the impact of CCBs, further advancing and accelerating systemic change toward a CE.

### Circular citizenship and consumption behaviors are related

**Figure 4:**
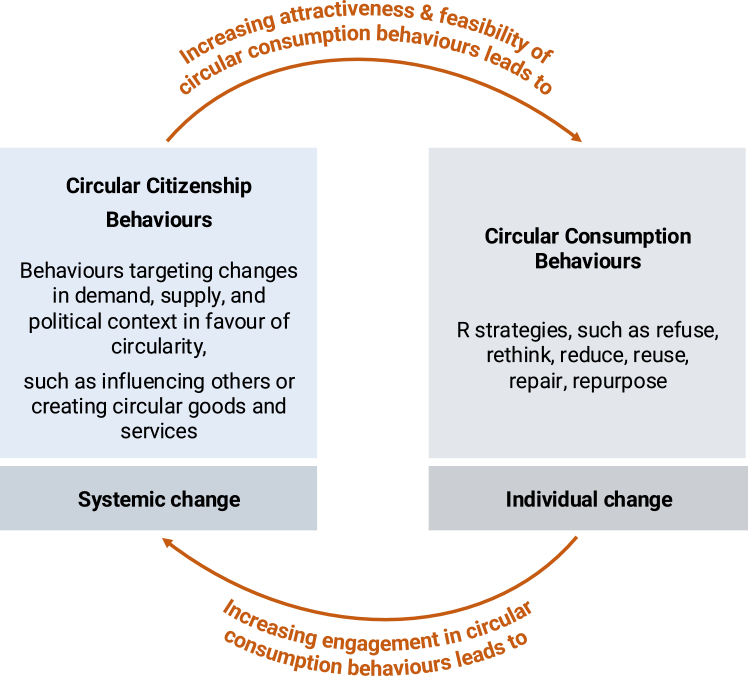
The relationship between circular citizenship and consumption behaviors

People can contribute to systemic change by engaging in CCBs through targeting the demand, supply, and political context. They can aim to influence other individuals, businesses, and governments to engage in the CE and create circular goods and services themselves, which can promote systemic change. Through such systemic change, engaging in circular consumption behaviors is likely to become more attractive and feasible. Therefore, circular consumption behaviors are more likely to be adopted, which demonstrates the impact that CCBs might have. At the same time, by engaging in circular consumption behaviors, people may influence others by serving as role models or establishing new social norms for behaviors, which can, in turn, promote circular demand. This implies that circular citizenship and consumption behaviors can mutually influence each other and might create an even greater impact through feedback loops. Figure 4 depicts this influence and the level of change achieved by circular citizenship and consumption behaviors.

## Significance of circular citizenship behaviors to promote systemic change

Our integrative SCCA framework for individuals’ behaviors to advance systemic change acknowledges that individuals are not merely consumers but are an active part of the societal system, which they can influence and shape.^42^^,^^113^ As such, our SCCA framework addresses criticism by various scholars about the current focal points of the CE. First, CCBs make it more likely that human needs and social demands are adequately addressed, thereby acknowledging calls for greater attention to social equity and solidarity in the transition to a CE.^8^^,^^38^^,^^43^^,^^44^^,^^114^^,^^115^^,^^116^^,^^117^ Second, the SCCA framework includes actions that foster civic engagement that enable citizens to play a stronger role in creating their future,^8^^,^^37^^,^^113^ fostering democratic and inclusive development and decision-making processes.^8^ Third, current CE approaches are sometimes seen as trying to address problems with the same systems that have caused these problems, while more profound change is seen as necessary for a transformation toward circularity.^38^^,^^41^^,^^43^

The SCCA framework presents behaviors promoting systemic and, thus, transformative change. More specifically, the CCBs included in the SCCA framework indicate that individuals are not powerless when they face barriers to acting but can engage in a wide variety of actions targeting different actors that could accelerate systemic change. This, in turn, is likely to promote circular consumption as it is likely to become more feasible, attractive, and normative. As such, CCB may have a large-scale impact by accelerating systemic change. Overlooking CCBs might, therefore, leave much potential untapped. Understanding CCBs is therefore critical for getting more comprehensive and accurate insights into what people (can) due to support the CE. Indeed, an individual might not engage in a particular circular consumption behavior but still significantly contribute to the CE by, for example, communicating about circularity to friends and family, protesting, and signing petitions. The SCCA framework provides an integrative framework for citizenship behaviors, integrating different research strands and domains, organizing and grouping them based on the actors targeted, and introducing them into the field of CE research.

Recognizing CCBs signals that the behavioral scope of individuals to support the CE is broader than solely changing their consumption. Specifically, it shows that individuals can also take action to target systemic change that, in turn, can facilitate and enable (more) circular consumption, which can, in the end, be highly impactful. Being aware of the range of behavioral options to support the CE is a prerequisite for citizens to engage in and promote these behaviors, and thus, a first step toward more engagement. The SCCA framework is the first to show this potential.

Importantly, CCBs show that citizens do not need to adopt a passive stance, awaiting actions from other actors to enable the CE when they face barriers that impede circular consumption. Rather, through CCBs, they can increase the likelihood that other actors act to remove barriers to circular consumption. For example, someone who wants to repair their phone but is not able to do so because they cannot find a store near them can engage in CCBs to eliminate this barrier in the future. CCBs also enable citizens to voice support and demands for more circularity regarding topics that are not directly related to consumption, such as circularity in business-to-business products and services, which supports the CE more broadly. This would facilitate the actions of many and thereby have a large impact.

Calls for system change are increasing.^118^^,^^119^^,^^120^ The concept of the CE demonstrates what an aspired system of our economy can look like. However, typically, calls for system change do not consider how individuals can contribute to it. Rather, it is assumed that systemic change means not to bother citizens, which implies that their potential contribution is not being utilized. CCBs increase our understanding of how citizens can play a critical role in transforming the current system into the CE. As demonstrated in the sections on the specific pathways of CCBs, real-world examples show that CCBs are feasible and actionable and can, indeed, promote systemic change.

The SCCA framework can be applied in practice by making citizens, organizations, and other societal groups aware of the many actions citizens can take to promote systemic change, and understanding how to promote CCBs, which would remove key barriers people face when they want to engage in circular behaviors and enable people to act in line with what they find important (as people generally care about and want to protect nature and the environment^121^). Our framework enhances the understanding that citizens do not have to wait until institutions and organizations change, but that citizens can take action to accelerate systemic change.

The SCCA framework focuses on how citizens can contribute to systemic changes toward a CE. Similar citizenship behaviors should promote systemic changes to address other environmental problems as well. Hence, our framework lays the foundation for organizing our thinking about citizenship behaviors that aim to reduce environmental problems more generally.

## Research agenda: Understanding circular consumption and citizenship behaviors

**Figure 5:**
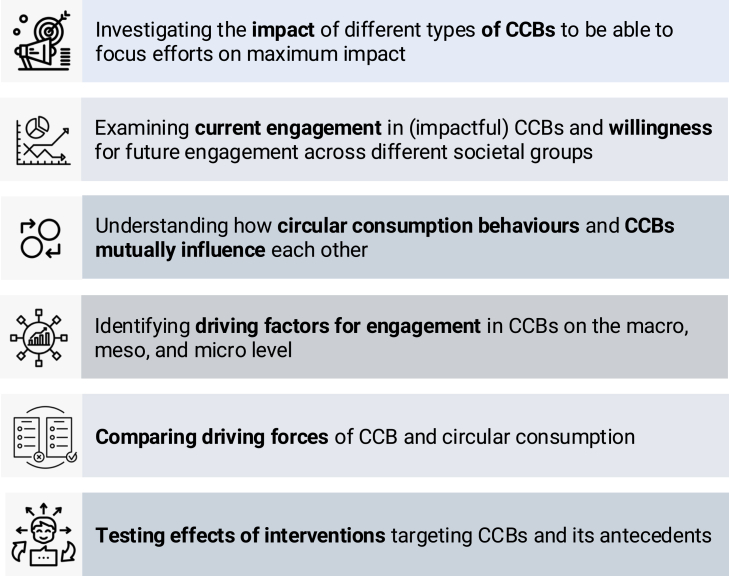
Future research directions

Earlier, we emphasized the significance of CCBs next to circular consumption behaviors. This opens up critical questions for future research (for an overview, see Figure 5).

First, future studies can examine the impact of different types of CCBs on systemic change, which can help focus efforts for maximum impact.

We need to understand current engagement in (impactful) CCBs and willingness to do so in the future across different groups in society (see literature on behavioral plasticity^122^^,^^123^). Some initial studies indicate that currently, only a few people seem to engage in CCBs. For example, less than 2% of UK residents indicated having taken part in protests, and less than 5% regularly wrote to politicians about environmental issues in 2022.^35^ Future studies can compare different levels of engagement across countries and cultures.

Research could also examine how circular consumption and citizenship behaviors influence each other. For example, when systemic barriers hinder circular consumption behaviors, individuals might engage in CCBs to promote systemic changes that would enable circular consumption. Understanding when and how circular consumption and citizenship behaviors reinforce each other can help maximize their combined impact.

Next, future studies can examine which factors drive CCBs, including macro-, meso-, and micro-level factors. For instance, on a macro level, autocratic or restrictive regimes may suppress behaviors like voting or protesting, and cultural values might influence social norms around circularity.^124^^,^^125^ Meso-level factors, such as organizational hierarchies, may inhibit CCB. Further, strong social norms for engagement may encourage, while the fear of social disapproval may discourage engagement in CCBs.^126^ Individuals’ characteristics, such as their values, beliefs, or financial resources, could also shape engagement in CCBs. Prominent theoretical models to explain pro-environmental actions, including the COM-B model,^127^ the theory of planned behavior,^128^ and the carbon capability framework,^36^^,^^40^^,^^45^ emphasize individuals’ capabilities (e.g., knowledge, cognitive, and behavioral skills) and opportunities (e.g., available infrastructure), which can affect how difficult citizens perceive a behavior to be, and motivation to engage in a behavior. Other theories, including the norm activation model,^65^ the value-belief-norm theory (VBN),^47^ the social identity theory,^129^^,^^130^ and the value-identity-personal norm model,^131^ highlight the role of values, identities, beliefs, and norms. Future studies can examine which of these models is most successful in explaining different types of CCBs.

Macro-, meso-, and micro-level factors may influence each other. The wider societal, political, and economic system can affect an individual’s opportunities, capabilities and motivation.^35^^,^^45^ Barriers on a macro-level are likely to affect perceived difficulty of the behavior, as reflected in self-efficacy—the perception that one can engage in a behavior. For example, in a country with an autocratic government, CCBs may be very difficult, risky, or even impossible, and therefore less likely compared to under a democratic government. Similarly, people may not feel capable of speaking out in very hierarchical organizations. Very low affluence or seeing CCBs as time-intensive or effortful^132^ may also result in high perceived behavioral difficulty and low self-efficacy. In such cases, people may not engage in CCB because of high perceived difficulty and low self-efficacy, resulting from serious political and organizational barriers or limited resources.

**Figure 6:**
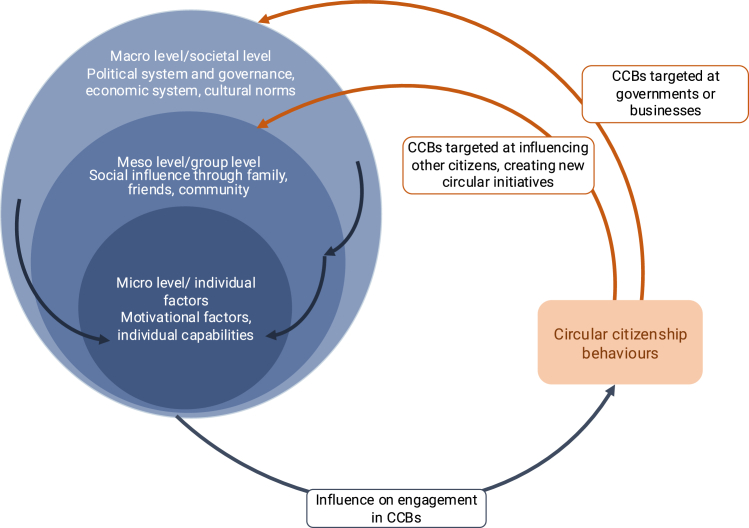
Dynamic influence between societal, group-level, and individual factors and circular citizenship behaviors

CCBs not only depend on systemic factors but, in turn, are likely to also influence them as they are aimed at promoting systemic change. For example, CCBs may promote changes in infrastructure offered by governments and businesses (macro level) or shift social norms (meso level), reinforcing systemic change (cf. Whitmarsh and colleagues^36^). The interplay between influencing factors and CCBs is illustrated in Figure 6.

The question remains whether similar (or different) factors drive the broad range of CCBs, including creating circular goods and services or influencing other individuals. While some factors likely affect a range of CCBs, others may influence only specific behaviors. For example, biospheric and altruistic values relate to diverse pro-environmental behaviors^87^ and may thus also affect various CCBs. Social norms may more strongly affect visible CCBs (such as protesting) than less visible actions (such as signing an online petition).

Further, we need to explore whether similar and/or different factors promote circular consumption and citizenship behaviors. The VBN theory^47^ assumes that similar factors, such as values, problem awareness, and personal norms, shape diverse pro-environmental behavior, including environmental citizenship and consumption. Various studies indeed indicate that the VBN theory can explain behaviors similar to CCBs, such as environmental activism, policy support and environmental citizenship behaviors, organizational behaviors, and boycotting, as well as circular consumption, e.g., circular consumption in the apparel industry, reducing car use or switching to an electric car, buying products made from recycled materials, or conserving energy.^49^^,^^133^^,^^134^^,^^135^^,^^136^^,^^137^^,^^138^^,^^139^^,^^140^^,^^141^ These behaviors can be categorized as *reduce*, *reuse*, and *recycle* strategies of circular consumption. The findings suggest that similar variables may be related to circular consumption and citizenship behaviors, though the strength may vary between behaviors.

Strategies can be implemented that encourage CCBs by targeting key antecedents, strengthening motivation, and removing barriers to act. Identifying determinants that influence both circular citizenship and consumption behaviors allows for developing interventions to promote systemic change as well as individual behavior change. Strategies to promote CCBs will be more effective when they target their main determinants.^142^ Future studies are needed to test which interventions are most effective in targeting key antecedents and barriers of CCBs and in encouraging such behaviors.

## Conclusion

We argued that it is critical to consider CCBs next to circular consumption behaviors, recognizing individuals’ full behavioral repertoire to contribute to a CE. We identified three pathways of CCBs to achieve systemic changes: encouraging changes in the demand toward more circularity by influencing other consumers, promoting improvements in the supply of circular goods and services by influencing businesses, and pressing for a political context that facilitates circularity by influencing governments. To achieve systemic changes, citizens can urge these other actors to engage in the CE. Within the three pathways, we identified a wide range of CCBs that could promote systemic change. These behaviors have mostly been overlooked in the literature on a CE so far, which is surprising as systemic changes are critical and likely enable consumer behavior. Future studies are needed to understand what enables and motivates people to engage in different types of circular behaviors—circular citizenship and consumption behaviors—and how such behaviors can best be promoted. CCBs acknowledge that individuals can have much more power than they might think and can accelerate the CE by urging others to act, which will increase the likelihood of much-needed systemic change. We presented a research agenda to better understand the antecedents and consequences of CCBs, as to better understand how to facilitate systemic change to create a more circular economy and reduce environmental problems and resource use.

## Acknowledgments

This research is part of the CircEUlar project, funded by the European Union's Horizon Europe Framework program under EC grant agreement 101056810.

## Author contributions

I.M.P. wrote the manuscript and prepared the table and figures, supervised by E.v.d.W. and L.S. All authors reviewed the manuscript.

## Declaration of interests

The authors declare no competing interests.

## References

[1] Pörtner H.-O., Roberts D.C., Tignor M., Poloczanska E.S., Mintenbeck K., Alegría A., Langsdorf S., Löschke S., Möller V., Okem A., IPCC (2022). Contribution of Working Group II to the Sixth Assessment Report of the Intergovernmental Panel on Climate Change.

[2] The World Bank (2023). https://www.worldbank.org/en/topic/pollution.

[3] Core Writing Team, Lee H., Romero J., IPCC (2023). Contribution of Working Groups I, II and III to the Sixth Assessment Report of the Intergovernmental Panel on Climate Change.

[4] Ellen MacArthur Foundation (2013).

[5] Mhatre P., Panchal R., Singh A., Bibyan S. (2021). A systematic literature review on the circular economy initiatives in the European Union. Sustain. Prod. Consum..

[6] Bocken N.M.P., De Pauw I., Bakker C., Van Der Grinten B. (2016). Product design and business model strategies for a circular economy. J. Indus. Prod. Eng..

[7] Circle Economy (2023). The Circularity Gap Report 2023.

[8] Clube R.K.M., Tennant M. (2023). What would a human-centred ‘social’ Circular Economy look like? Drawing from Max-Neef’s Human-Scale Development proposal. J. Cleaner Prod..

[9] Danvers S., Robertson J., Zutshi A. (2023). Conceptualizing How Collaboration Advances Circularity. Sustainability.

[10] Köhler J., Sönnichsen S.D., Beske-Jansen P. (2022). Towards a collaboration framework for circular economy: The role of dynamic capabilities and open innovation. Bus. Strat. Environ..

[11] Korsunova A., Horn S., Vainio A. (2021). Understanding circular economy in everyday life: Perceptions of young adults in the Finnish context. Sustain. Prod. Consum..

[12] Mishra J.L., Chiwenga K.D., Ali K. (2021). Collaboration as an enabler for circular economy: a case study of a developing country. Manage. Decisi..

[13] De Waal I.M. (2023). The Legal Transition towards a More Circular Electrical and Electronic Equipment Chain—A Case Study of The Netherlands. Sustainability.

[14] Geissdoerfer M., Pieroni M.P.P., Pigosso D.C.A., Soufani K. (2020). Circular business models: A review. J. Cleaner Prod..

[15] Nießen L., Bocken N. (2022). Circular business models. Ökologisches Wirtschaften - Fachzeitschrift.

[16] Urbinati A., Chiaroni D., Chiesa V. (2017). Towards a new taxonomy of circular economy business models. J. Cleaner Prod..

[17] Camacho-Otero J., Boks C., Pettersen I.N. (2018). Consumption in the Circular Economy: A Literature Review. Sustainability.

[18] Bocken N.M.P., Aagaard A. (2024). Business Model Innovation.

[19] Tukker A. (2015). Product services for a resource-efficient and circular economy – a review. J. Cleaner Prod..

[20] One Planet Network (2020). https://www.oneplanetnetwork.org/knowledge-centre/policies/circular-economy-promotion-law-peoples-republic-china.

[21] National Development and Reform Commission (2021). https://www.ndrc.gov.cn/xwdt/tzgg/202107/t20210707_1285530.html.

[22] Bleischwitz R., Yang M., Huang B., Xu X., Zhou J., McDowall W., Andrews-Speed P., Liu Z., Yong G. (2022). The circular economy in China: Achievements, challenges and potential implications for decarbonisation. Resour. Conserv. Recyc..

[23] European Commission (2020).

[24] Lüdeke-Freund F., Gold S., Bocken N.M.P. (2019). A Review and Typology of Circular Economy Business Model Patterns. J. Indus. Ecol..

[25] Selvefors A., Rexfelt O., Renström S., Strömberg H. (2019). Use to use – A user perspective on product circularity. J. Cleaner Prod..

[26] De Angelis R., Howard M., Miemczyk J. (2018). Supply chain management and the circular economy: towards the circular supply chain. Prod. Plan. Control.

[27] Govindan K., Hasanagic M. (2018). A systematic review on drivers, barriers, and practices towards circular economy: a supply chain perspective. Int. J. Prod. Res..

[28] Javaid A., Creutzig F., Bamberg S. (2020). Determinants of low-carbon transport mode adoption: systematic review of reviews. Environ. Res. Lett..

[29] Mundaca L., Román-Collado R., Cansino J.M. (2022). Assessing the impacts of social norms on low-carbon mobility options. Energy Policy.

[30] Silvestri A., Foudi S., Galarraga I. (2022). How to get commuters out of private cars? Exploring the role of perceived social impacts in mode choice in five European countries. Energy Res. Soc. Sci..

[31] Karlen C., Pagani A., Binder C.R. (2022). Obstacles and opportunities for reducing dwelling size to shrink the environmental footprint of housing: tenants’ residential preferences and housing choice. J. Housing Built Environ..

[32] Chan J.K.H., Zhang Y. (2021). Sharing Space: Urban Sharing, Sharing a Living Space, and Shared Social Spaces. Space Cult..

[33] Hashim M.Z., Awaluddin Z.L., A M R A., Sarkum S.A., Sholiha A.B., Aziz A.A. (2021). Architectural Design in Rethinking the Future of Co-Housing in Malaysia. JDBE.

[34] Kirchherr J., Reike D., Hekkert M. (2017). Conceptualizing the circular economy: An analysis of 114 definitions. Resour. Conserv. Recycling.

[35] Hampton S., Whitmarsh L. (2024). Carbon capability revisited: Theoretical developments and empirical evidence. Glob. Environ. Change.

[36] Whitmarsh L., Seyfang G., O’Neill S. (2011). Public engagement with carbon and climate change: To what extent is the public ‘carbon capable. Glob. Environ. Change.

[37] Hobson K., Brandão M., Lazarevic D., Finnveden G. (2020). Handbook of the Circular Economy.

[38] Hobson K., Hayes G., Jinnah S., Kashwan P., Konisky D.M., MacGregor S., Meyer J.M., Zito A.R. (2022). Trajectories in Environmental Politics.

[39] Moreau V., Sahakian M., Van Griethuysen P., Vuille F. (2017). Coming Full Circle: Why Social and Institutional Dimensions Matter for the Circular Economy. J. Indus. Ecol..

[40] Wei J., Chen H., Cui X., Long R. (2016). Carbon capability of urban residents and its structure: Evidence from a survey of Jiangsu Province in China. Appl. Energy.

[41] Calisto Friant M., Vermeulen W.J.V., Salomone R. (2024). Transition to a Sustainable Circular Society: More than Just Resource Efficiency. Circ. Econ. Sust..

[42] Jaeger-Erben M., Jensen C., Hofmann F., Zwiers J. (2021). There is no sustainable circular economy without a circular society. Resour. Conserv. Recycling.

[43] Leipold S., Weldner K., Hohl M. (2021). Do we need a ‘circular society’? Competing narratives of the circular economy in the French food sector. Ecol. Econ..

[44] Padilla-Rivera A., Do Carmo B.B.T., Arcese G., Merveille N. (2021). Social circular economy indicators: Selection through fuzzy delphi method. Sustain. Prod. Consum..

[45] Hampton S., Whitmarsh L. (2023). Choices for climate action: A review of the multiple roles individuals play. One Earth.

[46] Nielsen K.S., Nicholas K.A., Creutzig F., Dietz T., Stern P.C. (2021). The role of high-socioeconomic-status people in locking in or rapidly reducing energy-driven greenhouse gas emissions. Nat. Energy.

[47] Stern P.C. (2000). New Environmental Theories: Toward a Coherent Theory of Environmentally Significant Behavior. J. Soc. Issues.

[48] D’Arco M., Marino V. (2022). Environmental citizenship behavior and sustainability apps: an empirical investigation. Trop. Grassl..

[49] Kwame Yeboah F., Kaplowitz M. (2016). Explaining Energy Conservation and Environmental Citizenship Behaviors Using the Value-Belief-Norm Framework. Hum. Ecol. Rev..

[50] Yu T.-K., Lin F.-Y., Kao K.-Y., Chao C.-M., Yu T.-Y. (2019). An innovative environmental citizen behavior model: Recycling intention as climate change mitigation strategies. J. Environ. Manage..

[51] Aron A. (2022).

[52] Castiglione A., Brick C., Holden S., Miles-Urdan E., Aron A.R. (2022). Discovering the psychological building blocks underlying climate action—a longitudinal study of real-world activism. R. Soc. Open Sci..

[53] Marquart-Pyatt S.T. (2012). Explaining Environmental Activism Across Countries. Soc. Nat. Resour..

[54] Paço A., Gouveia Rodrigues R. (2016). Environmental activism and consumers’ perceived responsibility. Int. J. Cons. Stud..

[55] Ansell C., Gash A. (2008). Collaborative Governance in Theory and Practice. J. Public Admin. Res. Theory.

[56] Emerson K., Nabatchi T., Balogh S. (2012). An Integrative Framework for Collaborative Governance. J. Public Admin. Res. Theory.

[57] Greenwood S., Singer L., Willis W. (2021).

[58] Evans M., Terrey N., Stoker G., Evans M. (2016). Evidence-Based Policy Making in the Social Sciences: Methods That Matter.

[59] Re B., Magnani G. (2023). Value co-creation processes in the context of circular entrepreneurship: A quantitative study on born circular firms. J. Cleaner Prod..

[60] Ghinoi S., Silvestri F., Steiner B. (2020). The role of local stakeholders in disseminating knowledge for supporting the circular economy: a network analysis approach. Ecol. Econ..

[61] Kujala J., Heikkinen A., Blomberg A. (2023).

[62] Sijtsema S.J., Fogliano V., Hageman M. (2020). Tool to Support Citizen Participation and Multidisciplinarity in Food Innovation: Circular Food Design. Front. Sustain. Food Syst..

[63] Abrahamse W., Steg L. (2013). Social influence approaches to encourage resource conservation: A meta-analysis. Glob. Environ. Change.

[64] Geiger J.L., Steg L., Van Der Werff E., Ünal A.B. (2019). A meta-analysis of factors related to recycling. J. Environ. Psychol..

[65] Schwartz S.H. (1977). Advances in Experimental Social Psychology.

[66] Bandura A. (1986).

[67] Keizer K., Schultz P.W., Steg L., Groot J.I.M. (2018). Environmental Psychology.

[68] Deelmobiliteit.nu (n.d.). Is er aanbod van deelmobiliteit in jouw gemeente? https://deelmobiliteit.nu/.

[69] Milieucentraal (n.d.). Circulair: leven zonder afval. https://www.milieucentraal.nl/bewust-winkelen/duurzaam-delen-en-geven/circulair-leven-zonder-afval/.

[70] Jans L. (2021). Changing environmental behaviour from the bottom up: The formation of pro-environmental social identities. J. Environ. Psychol..

[71] Ruepert A.M., Keizer K., Steg L. (2017). The relationship between Corporate Environmental Responsibility, employees’ biospheric values and pro-environmental behaviour at work. J. Environ. Psychol..

[72] Ruepert A., Keizer K., Steg L., Maricchiolo F., Carrus G., Dumitru A., García Mira R., Stancu A., Moza D. (2016). Environmental considerations in the organizational context: A pathway to pro-environmental behaviour at work. Energy Res. Soc. Sci..

[73] Sharpe E., Ruepert A., Van Der Werff E., Steg L. (2022). Corporate environmental responsibility leads to more pro-environmental behavior at work by strengthening intrinsic pro-environmental motivation. One Earth.

[74] Kotler P. (1986). Prosumers: A New Type of Consumer. Futurist.

[75] Xie C., Bagozzi R.P., Troye S.V. (2008). Trying to prosume: toward a theory of consumers as co-creators of value. J. Acad. Market. Sci..

[76] Toffler A. (1980).

[77] Füller J., Hutter K., Faullant R. (2011). Why co-creation experience matters? Creative experience and its impact on the quantity and quality of creative contributions. R D Manage..

[78] Akhmedova A., Mas-Machuca M., Marimon F. (2020). Value co-creation in the sharing economy: The role of quality of service provided by peer. J. Cleaner Prod..

[79] Gentile C., Spiller N., Noci G. (2007). How to Sustain the Customer Experience. Eur. Manage. J..

[80] Piligrimienė Ž., Dovalienė A., Virvilaitė R. (2015). Consumer Engagement in Value Co-Creation: what Kind of Value it creates for Company?. Eng. Econ..

[81] Magnusson P.R., Matthing J., Kristensson P. (2003). Managing User Involvement in Service Innovation: Experiments with Innovating End Users. J. Serv. Res..

[82] Sawhney M., Verona G., Prandelli E. (2005). Collaborating to create: The Internet as a platform for customer engagement in product innovation. J. Inter. Market..

[83] Filieri R. (2013). Consumer co-creation and new product development: a case study in the food industry. Market. Intellig. Plan..

[84] Ki C., Chong S.M., Ha-Brookshire J.E. (2020). How fashion can achieve sustainable development through a circular economy and stakeholder engagement: A systematic literature review. Corp. Soc. Responsibil. Environ..

[85] Thomson I., Bebbington J. (2005). Social and environmental reporting in the UK: a pedagogic evaluation. Criti. Perspect. Accoun..

[86] Sheese K., Liu W., Teo T. (2014). Encyclopedia of Critical Psychology.

[87] Steg L., Gatersleben B., Murtagh N. (2023). Handbook on Pro-Environmental Behaviour Change.

[88] Bioy H., Stuart E. (2020).

[89] Beunderman M. (2024). Pensioenfonds ABP Komt Met Nieuwe Investeringsbeloften Tegemoet Aan Woningnood Bij Eigen Achterban.

[90] Seyfang G. (2009).

[91] Peerby Connect through Sharing: Lend, Borrow, and Rent Useful Things from People Near You. Peerby. https://www.peerby.com/en-nl.

[92] Jonas M., Nessel S., Tröger N. (2023).

[93] Sloot D., Jans L., Steg L. (2019). In it for the money, the environment, or the community? Motives for being involved in community energy initiatives. Glob. Environ. Change.

[94] Bradley K., Persson O. (2022). Community repair in the circular economy – fixing more than stuff. Local Environ..

[95] Milieudefensie et al. v. Royal Dutch Shell plc. (2019). Climate Change Litigation Databases. https://climatecasechart.com/non-us-case/milieudefensie-et-al-v-royal-dutch-shell-plc/.

[96] Izdebska O., Knieling J. (2020). Citizen involvement in waste management and circular economy in cities: Key elements for planning and implementation. Eur. Spatial Res. Policy.

[97] Lafortune G., Fuller G., Bermont Diaz L., Kloke-Lesch A., Koundouri P., Riccaboni A. (2022).

[98] How green politics are changing Europe (2021). BBC. https://www.bbc.com/news/world-europe-58910712.

[99] Douglas, R. (2023). Can Extinction Rebellion’s surprise success in the Netherlands be replicated? Waging nonviolence. https://wagingnonviolence.org/2023/12/extinction-rebellion-netherlands-a12-blockade-success/.

[100] Democracy Now! (2023). https://www.democracynow.org/2023/10/11/headlines/dutch_lawmakers_move_toward_phasing_out_fossil_fuel_subsidies_after_highway_blockade.

[101] COP28: Netherlands launches international coalition to phase out fossil fuel subsidies (2023). Government of the Netherlands. https://www.government.nl/latest/news/2023/12/09/cop28-netherlands-launches-international-coalition-to-phase-out-fossil-fuel-subsidies.

[102] NOS (2023). Jetten Leads International Group against Fossil Subsidies. NOS. https://nos.nl/artikel/2500946-jetten-voert-internationale-groep-tegen-fossiele-subsidies-aan.

[103] Pacheco, I. M., Van der Werff, E., and Steg, L. (2024). How circular is people’s behavior across Europe? CircEUlar. https://circeular.org/how-circular-is-peoples-behavior-across-europe/.

[104] Mackay C.M.L., Schmitt M.T., Lutz A.E., Mendel J. (2021). Recent developments in the social identity approach to the psychology of climate change. Curr. Opin. Psychol..

[105] Masson T., Fritsche I. (2021). We need climate change mitigation and climate change mitigation needs the ‘We’: a state-of-the-art review of social identity effects motivating climate change action. Curr. Opin. Behav. Sci..

[106] Campbell E., Kotcher J.E., Myers T., Munson S., Borth A.C., Rosenthal S.A., Leiserowitz A., Maibach E. (2023). The potential role of descriptive and dynamic norms in promoting climate change advocacy. Oxford Open Clim. Change.

[107] Sparkman G., Walton G.M. (2017). Dynamic Norms Promote Sustainable Behavior, Even if It Is Counternormative. Psychol. Sci..

[108] Sparkman G., Walton G.M. (2019). Witnessing change: Dynamic norms help resolve diverse barriers to personal change. J. Exp. Soc. Psychol..

[109] Centola D. (2018).

[110] Judge M., Bouman T., Steg L., Bolderdijk J.W. (2024). Accelerating social tipping points in sustainable behaviors: Insights from a dynamic model of moralized social change. One Earth.

[111] Mlakar, Ž. (2024). Tipping Towards Tomorrow: Understanding the Triggers and Dynamics of Sudden Societal Shifts. 10.33612/diss.1106999976.

[112] Van Der Werff E., Steg L., Ruepert A. (2021). My company is green, so am I: the relationship between perceived environmental responsibility of organisations and government, environmental self-identity, and pro-environmental behaviours. Energy Effic..

[113] Hobson K. (2016). Closing the loop or squaring the circle? Locating generative spaces for the circular economy. Prog. Hum. Geogr..

[114] Geissdoerfer M., Savaget P., Bocken N.M.P., Hultink E.J. (2017). The Circular Economy – A new sustainability paradigm?. J. Cleaner Prod..

[115] Hobson K., Lynch N. (2016). Diversifying and de-growing the circular economy: Radical social transformation in a resource-scarce world. Futures.

[116] Millar N., McLaughlin E., Börger T. (2019). The Circular Economy: Swings and Roundabouts?. Ecol. Econ..

[117] Murray A., Skene K., Haynes K. (2017). The Circular Economy: An Interdisciplinary Exploration of the Concept and Application in a Global Context. J. Bus. Ethics.

[118] Ballweg M., Bukow C., Delasalle F., Dixson-Declève S., Kloss B., Lewren I., Metzner J., Okatz J., Petit M., Pollich K. (2020).

[119] Cannon S.M. (2019).

[120] Monbiot G. (2023). https://www.bbc.co.uk/programmes/m001bd5p.

[121] Bouman T., Steg L. (2022). A spiral of (in)action: Empowering people to translate their values in climate action. One Earth.

[122] Koch J., Vringer K., Van Der Werff E., Wilting H., Steg L. (2024). Circular consumption to reduce environmental pressure: Potential of behavioural change in the Netherlands. Sustain. Prod. Consum..

[123] Moran D., Wood R., Hertwich E., Mattson K., Rodriguez J.F.D., Schanes K., Barrett J. (2020). Quantifying the potential for consumer-oriented policy to reduce European and foreign carbon emissions. Clim. Policy.

[124] Minton E.A., Spielmann N., Kahle L.R., Kim C.-H. (2018). The subjective norms of sustainable consumption: A cross-cultural exploration. J. Bus. Res..

[125] Saracevic S., Schlegelmilch B.B. (2021). The Impact of Social Norms on Pro-Environmental Behavior: A Systematic Literature Review of The Role of Culture and Self-Construal. Sustainability.

[126] Bolderdijk J.W., Cornelissen G. (2022). “How do you know someone’s vegan?” They won’t always tell you. An empirical test of the do-gooder’s dilemma. Appetite.

[127] Michie S., Van Stralen M.M., West R. (2011). The behaviour change wheel: A new method for characterising and designing behaviour change interventions. Implementation Sci..

[128] Ajzen I. (1991). The theory of planned behaviour. Organ. Behav. Hum. Decisi. Process..

[129] Tajfel H., Turner J., Williams J.A., Worchel S. (1986). The Social Psychology of Intergroup Relations.

[130] Tajfel H. (1978). Studies in the Social Psychology of Intergroup Relations.

[131] Van Der Werff E., Steg L. (2016). The psychology of participation and interest in smart energy systems: Comparing the value-belief-norm theory and the value-identity-personal norm model. Energy Res. Soc. Sci..

[132] Kaiser F.G., Schultz P.W. (2009). The Attitude–Behavior Relationship: A Test of Three Models of the Moderating Role of Behavioral Difficulty^1^. J. Appl. Soc. Pyschol..

[133] Chen M. (2015). An examination of the value-belief-norm theory model in predicting pro-environmental behaviour in Taiwan. Asian J Soc. Psycho..

[134] Gomes G.M., Moreira N., Bouman T., Ometto A.R., van der Werff E. (2022). Towards Circular Economy for More Sustainable Apparel Consumption: Testing the Value-Belief-Norm Theory in Brazil and in The Netherlands. Sustainability.

[135] Hein N. (2022). Factors Influencing the Purchase Intention for Recycled Products: Integrating Perceived Risk into Value-Belief-Norm Theory. Sustainability.

[136] Hiratsuka J., Perlaviciute G., Steg L. (2018). Testing VBN theory in Japan: Relationships between values, beliefs, norms, and acceptability and expected effects of a car pricing policy. Trans. Res. Part F Traffic Psychol. Behav..

[137] Jakovcevic A., Steg L. (2013). Sustainable transportation in Argentina: Values, beliefs, norms and car use reduction. Trans. Res. Part F Traffic Psychol. Behav..

[138] Jansson J., Marell A., Nordlund A. (2011). Exploring consumer adoption of a high involvement eco-innovation using value-belief-norm theory. J. Cons. Behav..

[139] Steg L., Dreijerink L., Abrahamse W. (2005). Factors influencing the acceptability of energy policies: A test of VBN theory. J. Environ. Psychol..

[140] Stern P.C., Dietz T., Abel T., Guagnano G.A., Kalof L. (1999). A Value-Belief-Norm Theory of Support for Social Movements: The Case of Environmentalism. Res. Hum. Ecol..

[141] Ünal A.B., Steg L., Granskaya J. (2019). “To support or not to support, that is the question”. Testing the VBN theory in predicting support for car use reduction policies in Russia. Trans. Res. Part A Policy Pract..

[142] Van Valkengoed A.M., Abrahamse W., Steg L. (2022). To select effective interventions for pro-environmental behaviour change, we need to consider determinants of behaviour. Nat. Hum. Behav..

